# Hypodermic Injections

**Published:** 1870-06

**Authors:** 


Hypodermic Injections.—It is now something more than ten years since the practice of introducing remedial agents under the skin for the relief of pain or the cure of disease, came into general use. It has passed through the various stages of suspicion, enthusiastic adoption, absurd extreme in its application, and finally of distrust in the minds of extremists, and now has taken its place as a most valuable method of exhibiting certain remedies, within proper limits, among the other resources of the profession. Occasionally some experimenter attempts to widen its sphere of usefulness by applying it to the general treatment of some disease, as lately, to the exhibition of mercury in syphilis, but the objections in the majority of cases exceed in number the advantages gained, and we find practically that the useful hypodermic administration of medicines is confined to those cases where remedies by the mouth are contraindicated from some cause, or where it is desirable to induce prompt action of a remedy. Generally it is applied for the relief of pain, and the promptness and facility with which anodynes produce their effects, when applied thus, is truly marvelous. At first it was supposed the dose thus given should be larger than when taken into the stomach, the directions published stating the quantity requisite to be from 50 to 100 per cent. greater than by the ordinary method. It is singular how long and persistently this opinion held ground, before the fact was recognized that owing to the more perfect and prompt absorption of the drug, when diffused in a concentrated solution in the subutaneous cellular tissue, than when taken into the stomach, a far smaller amount was needed to produce a decided impression than the common dose. The misapprehension of this fact, in the exhibition of the powerful narcotics, has led to fatal results repeatedly, and often to alarming toxic effects; indeed, it is surprising that death has not more frequently resulted from the grain doses of morphia which were fearlessly and constantly injected, producing profound narcotism lasting for from twelve to sixteen hours. It is with a shudder that we now recall the danger to which patients in our own experience have unwittingly been exposed, although, providentially, no accident has resulted.

On the other hand, it is surprising to what an extent severe pain obtunds the sensibility to powerful doses of narcotics thus administered. We recall a case of subacute inflammatory rheumatism in which the suffering was intense, and morphia, *per orem*, seemed to exercise no beneficial effect, even in large doses, but hypodermically a grain twice a day afforded quiet and comfort. A grain was the smallest quantity that would answer, as we only attained this dose after cautious experiment; for a few days as high as two and a half grains daily were required. This, it will be observed, is a far less quantity than in divided doses by the stomach would have been sufficient to produce the same constant sedation while the system was under the influence of such acute suffering.

